# Advancing Health Care With Digital Twins: Meta-Review of Applications and Implementation Challenges

**DOI:** 10.2196/69544

**Published:** 2025-02-19

**Authors:** Mickaël Ringeval, Faustin Armel Etindele Sosso, Martin Cousineau, Guy Paré

**Affiliations:** 1 HEC Montréal Montréal, QC Canada; 2 Centre Intégré Universitaire de Santé et de Services Sociaux du Nord-de-l’Île-de-Montréal Montréal, QC Canada

**Keywords:** digital twins, meta-review, health IT, applications, challenges, healthcare innovation, personalized medicine, operational efficiency

## Abstract

**Background:**

Digital twins (DTs) are digital representations of real-world systems, enabling advanced simulations, predictive modeling, and real-time optimization in various fields, including health care. Despite growing interest, the integration of DTs in health care faces challenges such as fragmented applications, ethical concerns, and barriers to adoption.

**Objective:**

This study systematically reviews the existing literature on DT applications in health care with three objectives: (1) to map primary applications, (2) to identify key challenges and limitations, and (3) to highlight gaps that can guide future research.

**Methods:**

A meta-review was conducted in a systematic fashion, adhering to PRISMA-ScR (Preferred Reporting Items for Systematic Reviews and Meta-Analyses extension for Scoping Reviews) guidelines, and included 25 literature reviews published between 2021 and 2024. The search encompassed 5 databases: PubMed, CINAHL, Web of Science, Embase, and PsycINFO. Thematic synthesis was used to categorize DT applications, stakeholders, and barriers to adoption.

**Results:**

A total of 3 primary DT applications in health care were identified: personalized medicine, operational efficiency, and medical research. While current applications, such as predictive diagnostics, patient-specific treatment simulations, and hospital resource optimization, remain in their early stages of development, they highlight the significant potential of DTs. Challenges include data quality, ethical issues, and socioeconomic barriers. This review also identified gaps in scalability, interoperability, and clinical validation.

**Conclusions:**

DTs hold transformative potential in health care, providing individualized care, operational optimization, and accelerated research. However, their adoption is hindered by technical, ethical, and financial barriers. Addressing these issues requires interdisciplinary collaboration, standardized protocols, and inclusive implementation strategies to ensure equitable access and meaningful impact.

## Introduction

Digital twins (DTs) are digital replicas of physical entities, processes, or systems dynamically updated with real-time data to reflect their real-world counterparts [1-4]. They enable predictive modeling, decision support, and personalized simulations in health care. DTs are often presented as 3D models or dashboards; for example, a cardiac DT might appear as a real-time, visual simulation of a patient’s heart. Clinicians can interact with the model by testing different treatment scenarios, such as adjusting medication dosages or simulating surgical interventions, and observe how the heart behaves—showing changes in function, blood flow, or potential complications in real time. This technology leverages sensors, analytics, and machine learning to enable monitoring, simulation, and predictive insights. Characterized by their ability to adapt to live data inputs, DTs facilitate real-time optimization, risk assessment, and enhanced decision-making across industries, from manufacturing to health care.

DTs offer transformative advantages over traditional tools by providing real-time insights and predictive analytics that enhance operational efficiency and decision-making. In sectors such as aerospace, DTs have reduced development times by up to 50% and significantly improved product quality [5]. As this technology advances, the global DT market is expected to grow rapidly, reaching US $110.1 billion by 2028, with an annual growth rate of 60% [6]. In health care alone, the market is valued at US $1.6 billion in 2023 and is projected to grow to US $21.1 billion by 2028, representing nearly 20% of the total DT market [7].

Health care applications of DTs offer the potential to simulate complex patient scenarios, support predictive diagnostics, and enable real-time, personalized treatment planning based on live health data [2]. Yet, as a new technology in the biomedical field, DT research in health care encompasses a wide range of clinical and operational applications at different stages of development, spanning from theoretical models to advanced experimental stages. This diversity—from individualized patient modeling to hospital workflow optimization—creates a fragmented body of research, where theoretical concepts and experimental models vary widely in sophistication. The variability in data sources, model design, and intended goals limits consensus on best practices and implementation pathways [8]. These factors highlight the need for a systematic review to synthesize the existing research, map out the various applications of DTs, and identify the gaps and challenges that remain.

The objective of this review is threefold. First, it seeks to provide a structured overview of the field, highlighting the diverse applications of DTs in health care. Second, it identifies and examines the barriers to DT adoption, along with potential strategies to address them. Third, it pinpoints gaps in existing knowledge to guide future research. The research questions addressed are as follows: (1) What are the primary applications of DTs in health care as identified in the extant literature? (2) What are the key challenges and limitations associated with implementing DTs in health care? (3) What gaps exist in current DT research in health care, and what are the implications for future research and practice?

## Methods

### Study Design

This meta-review was conducted and reported following the PRISMA-ScR (Preferred Reporting Items for Systematic Reviews and Meta-Analyses extension for Scoping Reviews) guidelines to ensure comprehensive reporting and methodological rigor [9]. The decision to use PRISMA-ScR reflects the emergent nature of DT research in health care, which requires a scoping approach to capture the breadth and diversity of this rapidly evolving field. Despite its emergent status, the field has already produced multiple literature reviews, indicating a growing maturity in this research stream. This apparent paradox reflects the rapid pace at which new knowledge is generated in DT research, necessitating both a broad mapping of the field and a deeper synthesis of existing findings. To address this dual need, we drew inspiration from the methodological approaches of Sarrami-Foroushani et al [10], incorporating elements of scoping meta-reviews. This hybrid approach allowed us to systematically map the literature while evaluating all existing reviews, enabling a comprehensive understanding of the field without restricting our analysis to systematic reviews alone.

### Search Strategy and Selection Criteria

A comprehensive literature search was conducted across multiple electronic databases without date restrictions, retrieving all available records up to May 15, 2024. The databases included PubMed, CINAHL, Web of Science, Embase, and PsycINFO. The search strategy and database selection were developed in collaboration with a professional librarian, who also executed the searches. The protocol for this review is registered on Open Science Framework [11].

Key search terms included “digital twin,” “intelligent twin,” and “mirror twin” combined with “review,” “systematic review,” and “meta-analysis.” To ensure the inclusion of all potentially relevant studies, the reference lists of selected papers were manually reviewed (snowballing). The complete search strategy for each database is provided in Multimedia Appendix 1.

We set inclusion criteria at the start of this study, allowing for potential revisions during the process. Papers were eligible for inclusion if they met the following criteria: (1) literature review paper (any type), (2) specifically about DT, (3) in the context of health care, and (4) accessible in full text in English or French. If a paper referenced DT applications in both health care and other settings, only the health care–related applications were considered.

All references were organized and deduplicated using EndNote (Clarivate), while Covidence (Veritas Health Innovation Ltd) was used for this study selection process.

### Study Selection

Two reviewers (MR and FAES) independently screened all titles and abstracts, with an initial screening of 30 references conducted to ensure a shared understanding of the selection criteria. This was followed by a full-text screening of selected studies to confirm eligibility.

The meta-review initially yielded 3377 references. After removing duplicates, we screened the titles and abstracts of 2921 references, retaining 65 for full-text review. Ultimately, 25 references were included in this review, with reasons for excluding others provided in the figure in the Results section.

### Data Extraction

Two authors (MR and FAES) independently extracted data from each of the included studies. Discrepancies were resolved through discussion. The following data were recorded: author, year, type of literature review (as reported), research questions, search period, included databases, final sample size, what DT models, DT application, potential or actual applications, DT provider, DT users, DT impacts, DT challenges, and main study results.

### Data Analysis

A mixed methods approach was used, combining descriptive and thematic analysis to map the landscape of DT technology in health care. Descriptive analysis was used initially to summarize the general characteristics of the included studies, covering the range of review types, publication years, and scope of DT applications.

As most reviews did not specify the type of review conducted (eg, scoping review and narrative review), we classified all reviews based on predefined criteria [12,13]. This classification process was conducted by one reviewer (MR) and verified by a second reviewer (FAES). Any discrepancies were resolved through discussion, ensuring a consistent and accurate categorization of review types.

## Results

### General Characteristics of the Included Reviews

**Figure 1:**
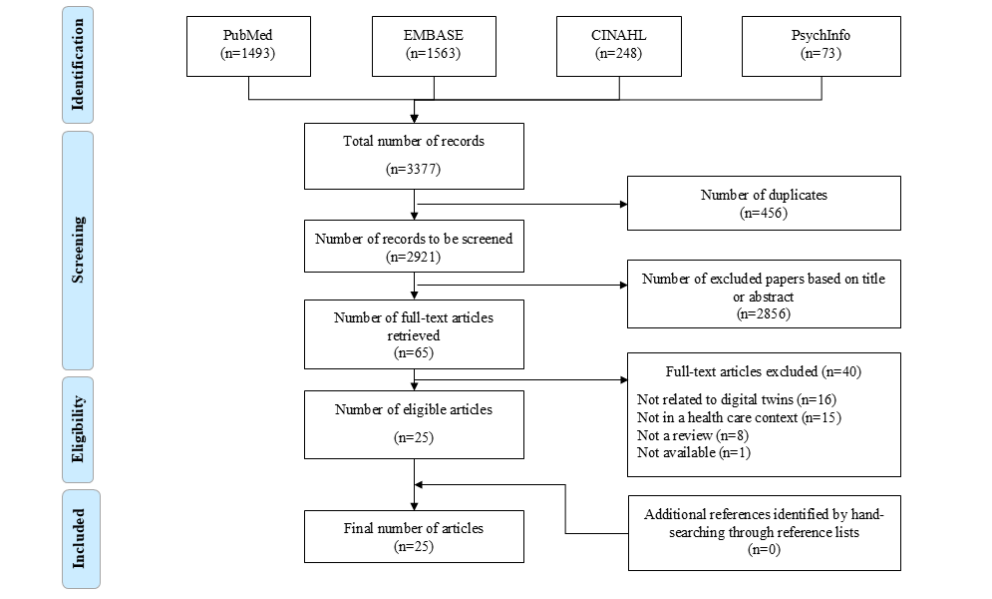
Flow diagram.

The included studies were published between 2021 and 2024 (Figure 1). Most of the reviews were categorized as scoping reviews (14/25, 56%) [1,2,14-25]. The remaining reviews included 6 narrative reviews (6/25, 24%) [26-31], 2 rapid reviews (2/25, 8%) [8,32], and 3 reviews for which a type could not be determined (3/25, 12%; see Multimedia Appendix 2 for the PRISMA-ScR checklist) [3,4,33].

### Studied Populations and Key Users of DTs

#### Targeted Populations

The reviews cover various populations, defined as the individuals or groups impacted by the outcomes of the DT application. For example, a DT designed for personalized medicine would mainly benefit patients, whether or not they were diagnosed with a specific disease.

Health care providers are a key target in 48% (12/25) of the reviews [2,8,19-23,25,29,30,32,33]. Similarly, patients in general are the focus of another 48% (12/25) of the reviews [2-4,8,14,19-21,27,28,30,32], underscoring the broad potential of DTs to impact patient care across diverse contexts. Beyond these general categories, 32% (8/25) of the reviews examine the general population [2,8,14,20,21,27-29], highlighting DT applications that extend to broader public health or societal benefits. Some studies further specify particular patient populations, such as those with cardiovascular diseases (7/25, 28%) [1,2,14,17,22,30,31] or multiple sclerosis (3/25, 12%) [20,24,25], suggesting targeted applications of DTs in managing chronic or complex conditions. Lastly, a small subset of reviews (2/25, 8%) does not specify any population for particular DT uses [30,32].

#### Users of DTs

DTs are used by various stakeholders, each benefiting uniquely from this technology. The primary users include patients [4,22-25,28,32,33], hospital management [8,14,19,21,28-30,32], and health care professionals, including clinicians [1,3,4,14,16,17,19,22-27,29-31,33], physicians [14,20,30,32], and researchers [2-4,14-17,19,20,22-25,27,29-31,33].

For patients, DTs provide tools to actively manage health conditions. For instance, patients with migraines [32] benefit from DT systems that integrate data from wearables and health records, offering real-time monitoring and predicting potential migraine triggers. This capability empowers patients to make informed decisions about lifestyle adjustments and treatment adherence, enhancing personal control over their condition by reducing the frequency or severity of migraine episodes.

In hospital management, DTs enhance operations across four main functions [8,14,21]. First, they improve safety by simulating emergency scenarios, allowing hospitals to identify risks and refine response protocols to protect patient well-being. Second, DTs streamline information management by consolidating real-time data from various sources, which improves communication and decision-making across departments. Third, in promoting health and well-being, DTs support personalized patient care by providing tools that adapt treatment protocols to individual data and forecast patient health trends, assisting health care professionals in delivering tailored interventions and preventive care. Lastly, DTs ensure efficient operational control by optimizing resource allocation, managing patient flow, and enabling predictive maintenance for equipment, which reduces patient wait times and enhances overall hospital efficiency.

DTs also serve as valuable tools for health care professionals, especially in supporting clinical decision-making and personalized treatment strategies. For complex cases such as cardiovascular and immune-mediated diseases, DTs enable clinicians to simulate patient-specific responses by integrating clinical, genetic, and environmental data. In cardiovascular care [1,17,19,20,30,31], DTs simulate heart function and disease progression, supporting tailored interventions based on individual patient profiles. By predicting and addressing patient-specific risks in real time, DTs enhance precision in medical practice, advancing the quality and personalization of care.

In sum, DTs support distinct user groups within health care. Patients and health care professionals, especially clinicians and physicians, benefit from DTs by providing precise, individualized care. Hospital management relies on DTs to enhance resource allocation, patient flow, and adaptability to changing demands. Clinicians and researchers leverage DTs to simulate virtual trials, advancing drug development and treatment refinement. These applications correspond to 3 main areas: personalized medicine, operational efficiency and resource management, and advancements in medical research and drug development.

#### Main Categories of DT Applications and Potential Benefits

DTs have emerged as a transformative tool in health care, offering tailored solutions across various applications. The following sections present 3 critical categories of DT applications, each accompanied by tables outlining the inputs, processes, and outputs relevant to each application. These tables provide a structured view of how concrete DT applications address specific health care challenges, optimize resources, and drive advancements in patient care and medical research.

#### Category 1: Customized Treatment and Care Optimization

One of the main applications of DTs in health care is customized treatment and care optimization (Table 1). DTs span various stages of development, from proof-of-concept models [1,8,14,17,19,20,28,30] to applications under active research and clinical testing [1,4,8,14-17,19,20,22,30-32]. Companies such as Dassault Systèmes, through their Living Heart Project [1,14,17,19,20], and Siemens [14,17,19,26] are at the forefront of these advancements. Other institutions have also developed some specific DTs, such as Medtronic with 3D cardiac maps [14,17] and Oklahoma State University with targeting tumor-only locations [14]. Some studies underscore the potential for DTs in complex areas [1,8,17,19,20,23,24,26,29,30,32], underscoring the nascent stage of this innovation in the field. Given its relative novelty, it is unsurprising that some review articles focus on potential applications while others concentrate on actual implementations.

**Table 1:** DT^a^ applications in customized treatment and care optimization.

Problem (and root causes)	Inputs	Processes	Outputs
Variability in patient physiology Delayed detection Limited predictive capability for disease progression	Data: Clinical data Genomic data Imaging data Biomarkers Lifestyle data Demographic data Sources: Electronic health record systems Disease registries Laboratory reports Wearable devices Surveys and questionnaires	Simulation: the DT can simulate a patient’s disease progression, the impact of treatment options, and various clinical scenarios, such as a sudden deterioration in condition or the patient’s response to a new medication. It can also model the effects of daily changes in lifestyle, nutrition, and medication adherence Optimization: based on simulation results, the DT can optimize personalized treatment plans, suggesting specific drug doses, dietary adjustments, or lifestyle interventions tailored to each patient’s condition and medical history Real-time decision-making: the DT can immediately alert health care providers to emerging risks such as arrhythmias or abnormal glucose levels, prompting timely interventions or adjustments in treatment	Real-time monitoring and prognosis Improved diagnosis Tailored health interventions and lifestyle guidance

^a^DT: digital twin.

The customized treatment and care optimization approach addresses key challenges in health care, including variability in patient physiology, delayed detection of disease progression, and limited predictive capabilities. By integrating diverse data inputs—such as clinical records, genomic information, imaging data, biomarkers, and lifestyle and demographic factors—sourced from electronic health record (EHR) systems, disease registries, and wearable devices [1,8,14,19,20,23,32,33], DTs enable comprehensive simulations of patient health profiles.

Through these simulations, DTs model disease progression, evaluate potential treatment options, and assess various clinical scenarios, such as predicting patient responses to new medications or lifestyle changes [1,2,4,14,17,20,22,24,28-30,32]. Beyond simulation, DTs support optimization by dynamically tailoring treatment plans to a patient’s evolving condition, which may include adjusting medication dosages, recommending dietary modifications, or suggesting lifestyle changes to improve outcomes [1,2,4,8,14,19,20,22-24,30,32].

Moreover, DTs support real-time decision-making by providing health care providers with alerts about emerging risks, such as abnormal glucose levels [1,14,30] or arrhythmias [31], enabling timely interventions. The outputs of this application—real-time monitoring, improved diagnosis, and personalized health interventions—underscore the potential of DTs to enhance personalized care and responsiveness in clinical practice.

#### Category 2: Operational Efficiency and Resource Management

Another significant category of DT applications in health care refers to operational efficiency and resource management (Table 2). DT technology serves as a valuable tool for optimizing hospital operations across various stages, from concept development [8,32] to active deployment [4,8,14,30]. For instance, GE HealthCare has developed a Command Center leveraging DTs to simulate and enhance patient flow in hospitals, including Johns Hopkins Hospital in Baltimore [8,14]. Similarly, organizations such as BioSecure and Siemens Healthineers have implemented DT-based solutions in health care settings [2,8] to optimize supply chain processes, improve response times for critical patients, and streamline workflows. While many initiatives demonstrate tangible benefits, others remain at the conceptual or exploratory stage, showcasing the potential for future DT applications in operational efficiency and resource management [21,23,32].

**Table 2:** DT^a^ applications in operational efficiency and resource management.

Problem (and root causes)	Inputs	Processes	Outputs
Unpredictable patient volumes Difficulty in dynamically adapting workflows in high-stakes situations Limited real-time operational insights that lead to inefficiencies and bottlenecks	Data: Patient data Historical trends Sources: Hospital information systems	Simulation: the DT can simulate hospital scenarios. By modeling these patterns, DTs can analyze how variations in patient flow affect wait times, bed availability, staffing requirements, and equipment usage, giving administrators foresight into potential bottlenecks Optimization: based on simulation results, the DT can recommend ideal staffing levels, identify underused or overbooked resources, and schedule maintenance during low-demand periods Real-time decision-making: the DT can suggest workflow shifts, such as rerouting noncritical cases, and improve discharge planning to free up beds efficiently	Optimized patient flow and resource Real-time adjustments in staffing and equipment usage based on patient arrivals Improved discharge planning and maintenance scheduling to ensure optimal bed availability and patient throughput Efficient crisis management strategies

^a^DT: digital twin.

The operational efficiency and resource management category addresses critical challenges such as unpredictable patient volumes, the difficulty of dynamically adapting workflows in critical situations, and the lack of real-time operational insights that often result in inefficiencies and bottlenecks. By leveraging inputs such as patient data and historical trends from hospital information systems [19,23], DTs can simulate various hospital scenarios. These simulations enable administrators to analyze how changes in patient flow impact key factors such as wait times, bed availability, staffing needs, and equipment use, thereby providing foresight into potential bottlenecks [2,8,14,19,23].

Beyond simulation, DTs enable optimization by recommending staffing adjustments, identifying underused or overburdened resources, and scheduling maintenance during low-demand periods [8,14,19,23,30,32]. Furthermore, DTs support real-time decision-making by suggesting workflow modifications, such as reallocating noncritical cases or expediting discharge planning to free up bed capacity [2,4,8,14,19,23]. The outputs of this category—optimized patient flow, real-time staffing and equipment adjustments, improved discharge planning, and effective crisis management strategies—highlight the significant potential of DTs to enhance resource use and operational responsiveness in health care facilities.

#### Category 3: Advancements in Medical Research and Drug Development

DT technology holds immense potential to transform medical research and drug development through in silico clinical trials, encompassing both prospective initiatives [3,17,19,30] and current applications (Table 3) [2-4,14,15,17,19,30]. For instance, collaborations involving Unlearn AI, Merck, Nvidia, and the University of Florida [14,19] are using DT models to simulate patient responses and design virtual clinical trials. These efforts enable the execution of single-arm studies, which are particularly valuable in scenarios where traditional randomized controlled trials may be unethical or impractical, such as rare diseases or high-risk populations. By simulating control groups, DTs help to reduce the need for placebo arms and contribute to minimizing adverse drug reactions [14,19,30]. Other applications of DTs focus on accelerating drug discovery and optimizing drug screening processes, offering promising avenues in pharmaceutical research [2-4,15,17].

**Table 3:** DT^a^ applications in advancements of medical research and drug development.

Problem (and root causes)	Inputs	Processes	Outputs
High costs and time demand of physical trials Ethical challenges in testing on human participants, especially in vulnerable populations Limited ability to predict individualized responses in diverse patient populations	Data: Clinical data Patient data Genomic data Imaging data Physiological data Sources: Electronic health records Wearable and remote monitoring devices Health databases Pharmaceutical and clinical trial data	Simulation: the DTs are used to simulate in silico clinical trials, allowing researchers to predict drug efficacy and safety across diverse patient profiles without the need for large-scale physical trials Optimization: with data from DTs, researchers can optimize trial parameters by adjusting dosages and selecting ideal patient cohorts. This approach refines drug formulations and identifies optimal therapeutic protocols, reducing unnecessary risks and costs associated with traditional clinical trials Real-time decision-making: If adverse reactions are predicted, DTs can recommend alternative treatment strategies, improving patient safety and the trial’s overall effectiveness	Synthetic control groups to minimize physical trials and enhance safety Predictive modeling of drug responses and adverse reactions, allowing for more accurate and efficient trials Simulations for drug efficacy testing, reducing the need for early-stage in-person trials Virtualized, patient-specific trials that allow researchers to adjust dosages and predict patient outcomes

^a^DT: digital twin.

This category of DT applications addresses critical challenges in clinical trials, including the high costs and time demands of traditional physical trials, ethical concerns related to testing on human participants—particularly in vulnerable populations—and the limited capacity to predict individualized patient responses. By integrating diverse data inputs such as clinical, genomic, imaging, and physiological data from sources such as EHRs, health databases, and pharmaceutical trial data [2,3,30], DTs enable the simulation of clinical trials in digital environments.

These simulations allow researchers to evaluate early-stage drug efficacy and safety across diverse patient profiles without the need for large-scale physical trials. This approach reduces costs and enhances safety through synthetic control groups [2-4,14,17,19]. Beyond simulation, DTs enable the optimization of trial parameters by adjusting dosages, selecting ideal patient cohorts, refining drug formulations, and identifying optimal therapeutic protocols. These capabilities help minimize unnecessary risks and costs [2-4,14,15,19].

Additionally, DTs support real-time decision-making by predicting adverse reactions and suggesting alternative treatment strategies to improve both patient safety and the trial’s overall success [2,3,14,17,19]. The outputs of these applications—including synthetic control groups, predictive modeling of drug responses, and virtualized patient-specific trials—demonstrate how DTs can significantly enhance the efficiency, accuracy, and personalization of clinical trials, making them more adaptable to the needs of diverse patient populations.

#### Types of DT

The classification of DT technologies originates from industry practices (eg, [34]), where DTs are typically categorized based on the scale and scope of the entities they represent. This framework enables a clear distinction between DTs by their purpose and level of detail, ranging from individual components to larger systems and entire processes.

Component or part DTs represent the most granular level, simulating individual components within larger systems. Examples include specific cardiac functions, such as aortic aneurysm [19], coronary anatomy [31], and coronary vessels [17]. Additionally, they encompass representations of individual cognitive profiles [25]. Asset or product DTs replicate individual physical assets, such as organs or medical equipment, to provide insights into performance and maintenance needs. This category includes organs [1,3,14,16,17,19,20,22,24,26,30,31], such as the heart [1,14,17,19,20,30,31] or the brain [14], as well as, to a lesser extent, diseases and therapeutic equipment [14,33]. System or unit DTs focus on the interactions of multiple components within a system. Examples include models of the human body (IDs 4, 21, 22, and 25), patients in various health conditions [1-3,14,17,19-21,27-30,32], and intensive care unit systems [8,14]. Lastly, process DTs simulate entire workflows to optimize health care operations. These include broader infrastructure models such as buildings [29], health care systems [8], hospitals [8,19], and physical systems [23].

#### DT Implementation Challenges

The implementation of DT technology in health care organizations faces several critical challenges, which can be grouped into 3 main categories: data and model integrity; ethical, regulatory, and governance challenges; and implementation and socioeconomic disparities.

First, data and model integrity encompasses issues related to data quality, availability, and the robustness of DT models. Limited access to high-quality data, difficulties in data integration, and privacy concerns represent significant barriers to adoption. Additionally, computational power demands and scalability challenges further complicate implementation [1,2,4,8,14-21,23,24,26,27,29,32,33]. Ensuring model validation and reproducibility remains a pressing issue, as the absence of standardized methods and randomized controlled trials undermines the clinical credibility of DTs. Moreover, the need to enhance model complexity to better capture individual patient variations highlights the current technological limitations of DTs [3,16,19,22,26,28,31].

Second, ethical, regulatory, and governance challenges address ethical considerations and the regulatory frameworks surrounding the use of DTs. Persistent concerns about bias, fairness, and data ownership highlight the risk of exacerbating existing socioeconomic disparities. Ethical dilemmas arise regarding informed consent and data ownership, particularly in the context of data sharing and potential misuse [2,4,8,14,16,17,19,20,24,29]. Furthermore, regulatory frameworks for DTs remain underdeveloped, creating uncertainties in legal governance, intellectual property protection, and alignment with existing health care regulations [4,8,17-20,24].

Third, implementation and socioeconomic disparities address the challenges of integrating DTs into clinical practice and the socioeconomic barriers to widespread adoption. Health care professionals often express concerns about trust, transparency, and the risk of job displacement, contributing to resistance against DT implementation. Additionally, there is a pressing need for enhanced education and training for both health care providers and patients to bridge the knowledge gap between clinicians and data scientists [4,14,16,17,23,30,32]. On a broader scale, the socioeconomic impacts of DTs include the potential to deepen the digital divide, limit access to advanced technologies, and enable financial barriers such as cost and reimbursement issues, which hinder broader implementation [1,18-20].

## Discussion

### Principal Results

This review highlights the transformative potential of DT technology across 3 critical applications in health care: personalized medicine, operational efficiency, and medical research and drug development. While these advancements present significant opportunities, they also introduce challenges that require attention to fully realize the benefits of DTs.

In personalized care, DTs leverage diverse data sources—such as clinical records, genomic information, and real-time inputs from wearable devices—to create patient-specific health simulations. These simulations serve as a foundation for predictive modeling, enabling tailored interventions and optimized treatment plans, particularly in complex domains such as cardiovascular care and immune-mediated diseases. However, many applications remain at the proof-of-concept stage, underscoring the need for further research, data standardization, and validation efforts to transition from experimentation to widespread clinical adoption.

DTs enhance health care operations by simulating patient flow, resource allocation, and emergency scenarios, offering actionable insights to improve efficiency. For instance, GE HealthCare’s Command Center demonstrates how DTs can reduce bottlenecks, optimize staffing, and enhance patient experiences [13]. However, adoption remains limited due to resistance to change, significant computational demands, and challenges in integrating DTs into existing workflows. Overcoming these barriers will require targeted training and interdisciplinary collaboration to facilitate the operational implementation of DTs.

DTs are transforming medical research by facilitating digital clinical trials and predictive modeling of drug responses. These innovations can significantly reduce the cost and ethical concerns associated with traditional trials while improving trial efficiency and safety. For example, collaborations between Unlearn AI and Merck are leveraging synthetic control groups to accelerate drug development [18]. However, broader implementation in this domain is contingent upon addressing critical challenges, including ensuring data quality, achieving scalability, and navigating regulatory approval processes.

### Challenges and Opportunities in Advancing DT Technology in Health Care

#### Overview

While our findings highlight the significant promise of DTs in health care, they also reveal that most current applications remain in their infancy, with many still at the proof-of-concept or early implementation stage. These initial deployments, though limited in scope, demonstrate promising results and lay a solid foundation for future advancements. The potential applications of DTs extend well beyond these early efforts, offering innovative solutions to address some of health care’s most pressing challenges. However, overcoming key barriers is essential to making DT technology more accessible, trustworthy, and practical for health care providers, thereby paving the way for wider adoption and meaningful impact.

First, challenges related to data and model integrity represent a significant concern for DT technology in health care. Integrating large datasets from wearables, EHRs, and imaging systems involves complexities in standardization, quality control, and real-time synchronization [35]. Ensuring model accuracy and minimizing artificial intelligence biases, as a key technology supporting DTs, are critical to making DTs reliable for high-stakes health care applications [36]. Addressing these issues requires standardized frameworks and rigorous validation protocols. Legal frameworks such as the European Union’s General Data Protection Regulation could serve as a model for implementing robust data governance practices within DT technology. To minimize artificial intelligence biases, validated practices established in EHRs can be leveraged to enhance data quality and ensure patient information integrity. Further, transparent validation processes, akin to clinical trials for medical devices, would foster trust and ensure DT reliability in clinical settings.

Next, ethical, regulatory, and governance challenges present significant concerns, particularly as DTs rely on sensitive health data. Issues such as data privacy, ownership, and the potential for algorithmic bias must be addressed to avoid undermining public trust and exacerbating disparities in care [37]. Adapting regulatory frameworks from virtual reality and internet of things devices could establish standards for data ownership, informed consent, and transparency, thereby mitigating risks of unauthorized data use. Furthermore, aligning these standards with international ethical guidelines could enhance public confidence in DT technology. The establishment of independent monitoring organizations—such as the European Medicines Agency [38], or ICANN (Internet Corporation for Assigned Names and Numbers) [39]—could provide consistent oversight and accountability. Such organizations would play a critical role in ensuring the ethical and responsible deployment of DTs across health care systems.

Finally, socioeconomic barriers to DT implementation raise concerns about exacerbating existing disparities in health care [40]. The high costs of advanced technologies create substantial obstacles for low- and middle-income countries to access essential tools needed for health innovation and economic modernization [41]. Moreover, the monopolization of patents and restrictive trade agreements by Western countries limits these nations’ ability to develop local solutions [42]. Dependency on international loans often shifts focus toward debt repayment instead of strategic investments in health care infrastructure, further perpetuating inequality and widening the digital divide. Lessons learned from the implementation of EHRs and connected medical devices show that cost-sharing models and collaborative funding initiatives can promote more equitable access to DT technology [43]. Open-source tools and public-private partnerships involving technology companies, health care providers, and research institutions offer additional pathways to reduce costs and enhance accessibility. Additionally, targeted training programs for health care professionals can bridge the skills gap, facilitating the successful adoption of DTs across diverse health care settings [44,45].

#### Future Directions for DT Technology in Health Care

Expanding DT technology in health care opens several important avenues for future research. A key challenge is the lack of a universally accepted definition, particularly in health care. While most studies define the term consistently, some variability persists, reflecting broader inconsistencies in the field. This lack of consensus complicates efforts to synthesize findings and assess the maturity of DT applications. Recognized definitions, such as those by the National Academies [46] and Drummond and Gonsard [47], emphasize the dynamic, predictive, and decision-informing attributes that distinguish DTs from other digital tools, which set them apart from other digital tools. Achieving definitional clarity is foundational for advancing practical applications, as inconsistent definitions risk undermining coherence in design, validation, and implementation. Future research should prioritize refining and standardizing DT definitions to foster alignment between research and real-world needs, enabling trust among stakeholders and paving the way for innovative, targeted health care solutions.

Future research should prioritize the practical implementation of DTs through detailed case studies and real-world applications, demonstrating their feasibility and clinical impact. Transparency and explainability are equally critical for building clinicians’ trust in DT recommendations and ensuring they can effectively interpret these insights. Addressing challenges related to scalability and data standardization is essential for widespread adoption, alongside rigorous validation through real-world clinical trials to establish the accuracy, safety, and seamless integration of DT systems into health care infrastructures.

Ethical considerations, including AI biases and patient privacy, require innovative approaches to algorithm development and the refinement of data ownership and informed consent models. Moreover, research into the socioeconomic impacts of DTs and cost-effective implementation strategies is vital to ensure accessibility for underserved populations. Special attention should be given to low-resource settings, where barriers such as cost, infrastructure, and training may hinder adoption. Collaboration between private and governmental sectors can ensure DT technology benefits a diverse range of patients and institutions, paving the way for equitable and sustainable adoption.

### Limitations

This review has limitations that may affect the interpretation of its findings. As a meta-review, our analysis is constrained by the scope and depth of the included studies, which may not comprehensively capture all aspects of DT applications and developments in health care. Furthermore, the rapid growth of DT research in health care, particularly with significant contributions from private industry, limits the visibility of current implementation efforts. Industrial stakeholders often hesitate to publish detailed information about their proprietary DT technologies and methodologies, a challenge also observed during the COVID-19 pandemic [48]. This restricts access to real-world data, reducing insights that could deepen our understanding of DT advancements.

### Conclusions

Though still in their early-stage applications, DTs hold immense potential to revolutionize health care through personalized medicine, operational efficiency, and accelerated medical research. However, realizing this potential requires addressing key challenges related to data integrity, ethical concerns, and socioeconomic disparities. Strategic investments, supportive policies, and collaborative research initiatives are crucial to ensuring equitable development. With an inclusive approach, DTs can transition from a visionary concept to a practical and transformative tool that enhances health care outcomes for diverse populations.

## Appendix

Multimedia Appendix 1 Research queries.

Multimedia Appendix 2 PRISMA-ScR checklist.

## Abbreviations

DT: digital twin
EHR: electronic health record
ICANN: Internet Corporation for Assigned Names and Numbers
PRISMA-ScR: Preferred Reporting Items for Systematic Reviews and Meta-Analyses extension for Scoping Reviews
