# Supplementary material for: Advancing Health Care With Digital Twins: Meta-Review of Applications and Implementation Challenges
Source: J Med Internet Res. 2025 Feb 19;27:e69544. doi: 10.2196/69544 (PMC11888003; doi:10.2196/69544)
Supplement: Multimedia Appendix 1 [file jmir_v27i1e69544_app1.doc]

**PubMed:**

1. digital twin* [Title/Abstract]
2. intelligent twin*[Title/Abstract]
3. mirror twin*[Title/Abstract]
4. meta-analysis[Title/Abstract]
5. review[Title/Abstract]
6. systematic review[Title/Abstract]
7. #1-3 AND #4-6/OR

**CINAHL:**

| S3 | S1 AND S2 |
| --- | --- |
| S2 | ("systematic review*") OR meta-analysis OR PT (review*) |
| S1 | TX "digital twin*" OR TX "intelligent twin*" OR TX "mirror twin*" |

**EMBASE:**

#11. #8 AND #9 AND #10

#10. #7 AND #6

#9. #7 AND #5

#8. #7 AND #4

#7. #1 OR #2 OR #3

#6. review*.mp

#5. meta-analysis.mp OR meta analysis

#4. “systematic review” OR “systematic review*”.mp

#3. "mirror twin*".mp

#2. "intelligent twin*".mp

#1. digital twin/ or "digital twin*".mp

**PsycInfo:**

#1. "digital twin*".mp

#2. "intelligent twin*".mp

#3. "mirror twin*".mp

#4. “systematic review”.mp OR exp “Systematic Review”

#5. “meta-analysis”.mp OR exp Meta Analysis/

#6. review*.mp

#7. #1 OR #2 OR #3

#8. #7 AND #4

#9. #7 AND #5

#10. #7 AND #6

#11. #8 AND #9 AND #10
